# Receptor-Dependent Endocytosis Mediates α-Synuclein Oligomer Transport Into Red Blood Cells

**DOI:** 10.3389/fnagi.2022.899892

**Published:** 2022-05-20

**Authors:** Wei Li, Junya Hu, Xin Li, Zhe Lu, Xuying Li, Chaodong Wang, Shun Yu

**Affiliations:** ^1^Department of Neurobiology, Xuanwu Hospital of Capital Medical University, Beijing, China; ^2^Center of Parkinson’s Disease, Beijing Institute for Brain Disorders, Beijing, China; ^3^Department of Neurology, Xuanwu Hospital of Capital Medical University, Beijing, China; ^4^National Clinical Research Center for Geriatric Diseases, Beijing, China

**Keywords:** α-synuclein, transport, red blood cell, receptor-dependent endocytosis, synucleinopathy

## Abstract

Detection of oligomeric α-synuclein (o-α-Syn) in red blood cells (RBCs) has been shown to be promising in diagnosing Parkinson’s disease and other synucleinopathies. However, if RBC o-α-Syn derive from plasma and can reflect changes of plasma o-α-Syn remains unclear. In this study, synthetic o-α-Syn was intravenously injected into mice and dynamic changes in plasma and RBC o-α-Syn levels were investigated. Injection of o-α-Syn induced a temporary increase in plasma o-α-Syn levels, which then decreased to a relatively stable level. In contrast, levels of RBC o-α-Syn increased steadily and significantly. Besides, α-Syn-immunoreactive particles were observed in RBCs of the injected mice, suggesting that RBCs can actively take up and enrich o-α-Syn from plasma. Moreover, incubation of o-α-Syn with isolated RBCs at concentrations lower than those of endogenous o-α-Syn led to a time- and concentration-dependent o-α-Syn elevation in RBCs, which was impaired by lowering the temperature and treatment with proteinase K. The o-α-Syn accumulation in RBCs was also inhibited by specific inhibitors of receptor-dependent endocytosis, including dynamin- and clathrin-dependent endocytosis. The above results suggest that plasma o-α-Syn can be actively transported into RBCs via receptor-dependent endocytic pathways.

## Introduction

Aberrant α-synuclein (α-Syn) aggregation, which may be caused by genetic and environmental factors, plays a central role in the pathogenesis of synucleinopathies, including Parkinson’s disease (PD), dementia with Lewy bodies (DLB), and multiple system atrophy (MSA; Spillantini et al., 1997; Goedert, 2001; Stefanis, 2012; Villar-Piqué et al., 2016; Wong and Krainc, 2017; Mehra et al., 2019). In particular, soluble monomeric α-Syn (m-α-Syn) aggregates into insoluble fibrils, forming disease-specific pathological structures such as Lewy bodies and Lewy neurites in neurons of the brain with PD and DLB and glial cytoplasmic inclusions in oligodendrocytes of the brain with MSA (Baba et al., 1998). Although fibrillar α-Syn aggregates constitute the major component of the hallmark pathological structures, numerous cell and animal experiments have demonstrated that oligomers or protofibrils are toxic to neurons (Danzer et al., 2007; Winner et al., 2011). The critical role of α-Syn in the pathogenesis of synucleinopathies makes it an ideal diagnostic biomarker for these disorders.

Various studies have been conducted to measure the concentrations of different α-Syn forms in body fluids (Maitta et al., 2011; Bencs et al., 2020; O’Hara et al., 2020; Ganguly et al., 2021). Although most results showed an increase in oligomeric α-Syn (o-α-Syn) and phosphorylated α-Syn (p-α-Syn) levels in cerebrospinal fluid (CSF), plasma, and saliva of patients with PD compared with those in healthy controls, the values were variable (Foulds et al., 2011; Hansson et al., 2014; Atik et al., 2016; Fayyad et al., 2019). Measurement results of total α-Syn (t-α-Syn) in body fluids are inconsistent (Lee et al., 2006; Li et al., 2007; Duran et al., 2010; Shi et al., 2010; Goldman et al., 2018; Bougea et al., 2019). One of the major reasons for the lack of consistency and stability is attributed to hemolysis contamination, as red blood cells (RBCs) contain high α-Syn concentrations and a small amount of hemolysis causes the release of RBC α-Syn into plasma and CSF. A recent study reported that higher α-Syn levels in CSF are associated with increased blood contamination (Barkovits et al., 2020). Plasma, serum, and saliva samples used in diagnostic tests might also be affected by hemolysis contamination (Jonsson et al., 2013; Emelyanov et al., 2016; Bougea et al., 2019), which highlights the difficulty of quantifying α-Syn in body fluids. Thus, direct detection of RBC α-Syn is an option that might not only avoid hemolysis interference but also acquire relatively stable results owing to high α-Syn concentrations in RBCs.

Several studies have been conducted to detect RBC α-Syn, including t-α-Syn, o-α-Syn, p-α-Syn, and proteinase K-resistant α-Syn (PK-α-Syn; Wang et al., 2015; Liu et al., 2019; Tian et al., 2019; Li et al., 2021). The levels of these α-Syn forms are increased in the RBCs of patients with PD and MSA compared with those in healthy controls, yielding moderate to higher diagnostic power in discriminating patients from healthy controls. However, the origin of RBC α-Syn remains unclear. α-Syn has been shown to cross the blood-brain barrier in both the brain-to-blood and blood-to-brain directions (El-Agnaf et al., 2006; Sui et al., 2014; Matsumoto et al., 2017); α-Syn overexpression in the mouse brain induced a significant increase in p-α-Syn levels in the brain as well as a parallel increase in p-α-Syn levels in both plasma and RBCs, indicating that RBC α-Syn can reflect the changes in brain α-Syn levels (Li et al., 2019). However, because α-Syn concentration in plasma is 1000 times lower than that in RBCs (Foulds et al., 2011; Nuzhnyi et al., 2015; Wang et al., 2015; Tian et al., 2019), if and how the α-Syn in plasma is transported into RBCs remains to be elucidated.

Because o-α-Syn, a major toxic form of α-Syn to neurons, is significantly increased in patients with PD and MSA, in the present study, we focused on revealing potential mechanisms of o-α-Syn transport from plasma into RBCs. We found that o-α-Syn could be transported from plasma into RBCs via an active transport mediated by receptor-dependent endocytic pathways.

## Materials and Methods

### Animals

Six-week-old C57BL/6J male mice with the weight of 20 g were purchased from Charles River Laboratories and housed under a 12 h light/dark cycle with *ad libitum* access to food and water. The mice were divided into two groups; one group was intravenously injected with o-α-Syn and the other group was intravenously injected with the same volume of saline without o-α-Syn.

### Blood Samples

Human and mouse RBCs were used in this study. To detect the changes in o-α-Syn levels in the plasma and RBCs of mice, the mice were anesthetized with 3.0–3.5% isoflurane and 2% oxygen and the blood was collected from the inferior vena cava into an Eppendorf tube containing 1.8% ethylenediamine tetraacetic acid (EDTA) buffer (10% v/v, final; Kattula et al., 2018). The blood was centrifuged at 4°C and 1,500 × *g* for 15 min and separated plasma and RBCs were collected.

Human plasma and RBC samples were obtained from 69 healthy participants. The detailed exclusion criteria have been previously described (Li et al., 2021). To obtain plasma and RBC samples, 10 mL of venous blood was drawn into an EDTA-coated tube (1.8 mg EDTA per tube; BD, Franklin Lake, NJ, United States) and centrifuged at 4°C and 1,500 × *g* for 15 min. The plasma and RBC samples were separately collected, aliquoted, and stored at –80°C for subsequent measurements of o-α-Syn concentrations. Some of RBCs were suspended in Krebs–Henseleit (KH) buffer (118 mM NaCl, 4.7 mM KCl, 1.2 mM MgSO_4_, 25 mM NaHCO_3_, 11 mM glucose, 2.4 mM CaCl_2_, pH 7.4; Zhou et al., 2018) and immediately used for *in vitro* o-α-Syn transport studies.

### Preparation of α-Syn Oligomers

Recombinant human α-Syn and its oligomers were prepared according to the methods described previously methods (Li et al., 2020). In brief, pET-15b-NACP plasmids were transformed into Escherichia coli BL21 cells and the expressed α-Syn proteins were purified sequentially with ion exchange chromatography, hydrophobic chromatography, and reverse phase chromatography. To prepare o-α-Syn, the purified human α-Syn (5 mg/mL) was dissolved in 0.01 M phosphate-buffered saline (PBS, pH 7.4) and incubated by constant shaking at 37°C (650 rpm) for 7 days on an Eppendorf Thermomixer Comfort (Eppendorf AG 22331, Hamburg, Germany; Li et al., 2020). The protein aggregates were then centrifuged at 100,000 × *g* for 1 h to remove insoluble α-Syn fibrils and the supernatant containing the mixture of m-α-Syn and o-α-Syn was collected. Soluble o-α-Syn in the mixture was separated using sodium dodecyl sulfate-polyacrylamide gel electrophoresis (SDS-PAGE) and recovered from the gel using a Micro Protein Recovery Kit (Sangon Biotech, C500062, Shanghai, China) according to the manufacturer’s protocol. The protein concentrations were determined using bicinchoninic acid protein assay kit (Thermo Scientific, 23235 Waltham, MA, United States) and purified oligomers were aliquoted and stored at –80°C.

### Transmission Electron Microscopy

Purified o-α-Syn were placed on the copper grids coated with Formvar. The samples on the grids were washed thrice with distilled water and stained with 2% uranyl acetate. Excess staining was removed by blotting and air drying. The samples were then visualized under transmission electron microscope (Jeol, JEM-2100, Japan; Yu et al., 2019).

### *In vivo* Oligomeric α-Synuclein Transport Study

To observe o-α-Syn transport from plasma into RBCs *in vivo*, 100 μL of 0.9% (w/v) saline containing 20 μg/mL of purified o-α-Syn was injected into the mouse tail veins. Control mice were administered an equal volume of o-α-Syn-free saline. Whole intravenous blood was collected from the mice at 0, 1, 6, and 12 h after o-α-Syn injection and plasma and RBCs were collected for measuring the changes in o-α-Syn levels using ELISA. In addition, o-α-Syn accumulation in RBCs was also detected using Western blot and immunofluorescence labeling 12 h after injection.

### *In vitro* Oligomeric α-Synuclein Transport Study

Freshly isolated human RBCs were washed thrice with KH buffer and then diluted with KH buffer to a 2% hematocrit in a total volume of 500 μL. The RBCs were incubated in a 24-well plate at 37°C with 2 μg/mL of o-α-Syn for different time periods (2–8 h) or with different o-α-Syn concentrations (0.02–2 μg/mL) for 8 h. After incubation, the RBCs were washed thrice with PBS and intracellular o-α-Syn levels were detected using enzyme-linked immunosorbent assay (ELISA), Western blot, and immunofluorescence.

### Effects of Temperature and Proteinase K on Oligomeric α-Synuclein Transport

RBCs were incubated with 2 μg/mL of o-α-Syn at 4 and 37°C for different time periods (4–12 h) to test the effect of temperature on o-α-Syn transport. In another experiment, human RBCs were pretreated for 10 min with KH buffer containing different PK (Merck kGaA, 1245680100, Darmstadt, Germany) concentrations (0.1–2 μg/mL). The RBCs were washed thrice with PBS to stop the reaction before incubation with 2 μg/mL o-α-Syn for 8 h at 37°C. After incubation, the RBCs were washed thrice with PBS and intracellular o-α-Syn levels were measured using ELISA and Western blot.

### Effects of Endocytosis Inhibitors on Oligomeric α-Synuclein Transport

Human RBCs were pretreated for 30 min at 37°C with KH buffer containing different endocytosis inhibitors before incubation with 2 μg/mL of o-α-Syn for 8 h. The inhibitors and their concentrations used were as follows: dynasore (Sigma-Aldrich, D7693, St. Louis, MO, United States), a specific inhibitor of dynamin-dependent endocytosis, 10 and 20 μM; pitstop 2 (Sigma-Aldrich, SML1169, St. Louis, MO, United States), a specific inhibitor of clathrin-mediated endocytosis, 10 and 20 μM. The specific dose of inhibitor has been reported previously (Macia et al., 2006; Park et al., 2009; Dutta et al., 2012), and the effect of inhibition on the endocytosis was assessed by observing the transmission of fluorescent labeled transferrin (Thermo Fisher, T-2871, Waltham, MA, United States) after the pretreatment of 20 μM dynasore and pitstop 2. After incubation, the RBCs were washed thrice to remove residual o-α-Syn and ELISA and Western blot were performed.

### Immunofluorescence

RBCs isolated from mouse blood samples and human RBCs incubated with or without o-α-Syn *in vitro* were washed thrice with PBS and fixed with 0.01% glutaraldehyde (10%, v/v). The RBCs were then treated with 0.001% Triton X-100 in PBS (10%, v/v). After blocking with 5% normal goat serum, the cells were incubated with 3D5 mouse monoclonal anti-human α-Syn antibody (RRID:AB_2315787, 1:1000) overnight at 4°C, followed by 2 h incubation at 25°C with the Alexa Fluor 488^®^ -conjugated goat anti-mouse immunoglobulin (Ig)G (Thermo Fisher, A-10680, Waltham, MA, United States, 1:500; Yu et al., 2007; Chen et al., 2017). After washing thrice with PBS, the cells were observed under a fluorescence microscope (Nikon, Ni-U, Japan) and confocal microscope (STELLARIS, Leica, Germany).

### Western Blot Analysis

To measure o-α-Syn concentrations in RBCs, the RBCs were lysed by two sequential freeze (–80°C) and thaw (room temperature, 25°C) cycles. RBC lysates were resuspended in PBS. RBC samples (50 μg proteins/lane) were separated on a 12.5% SDS-PAGE gel and transferred onto a 0.45 μm polyvinylidene fluoride membrane (Millipore Corp, IPVH00010, Boston, MA, United States). The membranes were blocked for 1 h with 5% non-fat milk in triethanolamine buffered saline containing 0.05% Tween-20 (TBST) and incubated with 3D5 mouse monoclonal antibody (1: 5000) at 4°C overnight. After washing with TBST, the membranes were incubated with horseradish peroxidase (HRP)-conjugated goat anti-mouse IgG (Thermo Fisher, 31430, Waltham, MA, United States, 1:5000) for 1 h at 37°C. The blots were visualized using Immobilon Western Chemiluminescent HRP Substrate (Merck Millipore, WBKLS, Billerica, MA, United States) and the optical density was measured using the Image J software. Hemoglobin (Hb) was used as control blots since Hb levels are very stable in RBCs.

### Enzyme-Linked Immunosorbent Assay

O-α-Syn levels in plasma and RBCs were measured using the ELISA method described before (Liu et al., 2015). To detect o-α-Syn changes in RBCs, RBCs were lysed as whole cell lysates and in some experiments as membrane and cytosolic fractions according to the method described before (Alvarez-Llamas et al., 2013). To detect o-α-Syn, non-biotinylated and biotinylated 3D5 mouse monoclonal antibodies were used for the capture and detection, respectively. Different concentrations of o-α-Syn standard solution and plasma and RBC samples were added to each well before the ELISA plate was incubated with the detection antibody. Then, the plate was incubated with 100 μL of streptavidin alkaline phosphatase (Vector Laboratories, SA-5100, Burlingame, CA, United States) diluted 1: 20,000 in blocking buffer and reacted with the enzyme substrate p-nitrophenyl phosphate (Sigma-Aldrich, 487655, St. Louis, MO, United States). The absorbance at 405 nm was read using a Multiskan MK3 microplate reader (Thermo Fisher Scientific, Waltham, MA, United States). The samples were measured in triplicates within the same assay and on the same day. The o-α-Syn concentrations were calculated according to the standard curve, and expressed as ng/mg when o-α-Syn concentrations in RBCs were compared in different treatments, and ng or μg/mL when o-α-Syn concentrations were compared between plasma and RBCs.

### Statistical Analysis

Statistical analyses were performed using the GraphPad Prism 8 software (GraphPad, San Diego, CA, United States). The levels of o-α-Syn in RBCs and plasma were expressed as means ± standard deviation (SD), and showed as dot plots to reflect the individual values. Student’s t-test was used for comparisons between two groups and one-way ANOVA with Tukey’s *post hoc* test was used for comparisons between multiple groups. The influence of incubation time and different PK concentrations on the transport of o-α-Syn was analyzed using Pearson’s correlation. Statistical significance was set at *p* < 0.05.

## Results

### Characterization of α-Syn Oligomers

**FIGURE 1:**
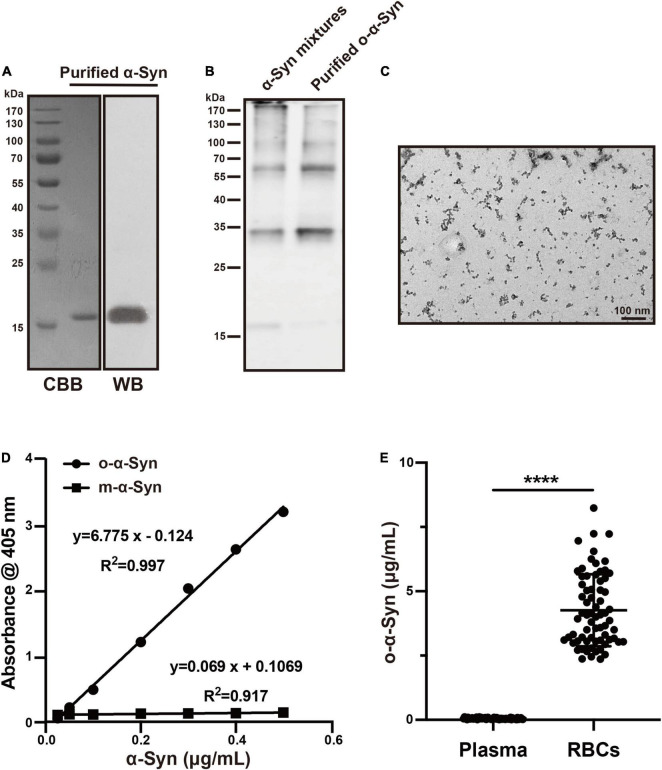
O-α-Syn characterization and its concentrations in plasma and Red Blood Cells (RBCs). **(A)** Recombinant m-α-Syn showed a single band at 17 kDa in CBB staining and Western blot analysis. **(B)** Monomers (17 kDa) and oligomers (34–170 kDa) was in non-purified samples; oligomers (34–170 kDa) was in purified samples. **(C)** Transmission electron microscopic analysis of the purified o-α-Syn, which presented spherical particles. **(D)** M-α-Syn and o-α-Syn were detected using o-α-Syn-specific ELISA. A linear correlation between absorbance at 405 nm and α-Syn concentration was observed for o-α-Syn (*R*^2^ = 0.997) but not for m-α-Syn. **(E)** O-α-Syn concentrations in RBCs were significantly higher than those in plasma (*n* = 69). CBB: Coomassie brilliant blue; WB: Western blot; ****p < 0.0001.

Purified recombinant human α-Syn presented a single band of 17 kDa corresponding to the molecular size of m-α-Syn in Coomassie brilliant blue (CBB) staining and Western blot analysis after SDS-PEGE (Figure 1A). Non-purified and purified o-α-Syn were also examined using Western blot analysis and o-α-Syn-specific ELISA. Western blot analysis revealed that the purified o-α-Syn samples contained only oligomers ranging from 34 to 170 kDa, corresponding to dimers, tetramers, hexamers, octamers and decamers (Figure 1B). In contrast, in the non-purified α-Syn mixture there was also monomeric α-Syn to be detected in addition to α-Syn oligomer. Purified o-α-Syn was also examined by transmission electron microscopy, which exhibited spherical aggregates (Figure 1C). No filaments were observed. The purified o-α-Syn was detected using o-α-Syn-specific ELISA. The results are shown in Figure 1D. A linear correlation between the absorbance at 405 nm and α-Syn concentration was observed only for o-α-Syn (*R*^2^ = 0.997, *p* < 0.001) and not for m-α-Syn.

### Normal Oligomeric α-Synuclein Concentrations in Plasma and Red Blood Cells

T-α-Syn concentration in human RBCs is reported to be 1000 times higher than that in plasma (Barbour et al., 2008); however, the difference between o-α-Syn concentrations in human RBCs and plasma remains unclear. Therefore, we measured o-α-Syn concentrations in human plasma and RBCs using o-α-Syn-specific ELISA method (Figure 1E). The results showed that the average o-α-Syn concentrations in RBCs and plasma were 4.263 ± 1.399 and 0.053 ± 0.028 μg/mL, respectively. This difference was statistically significant (*n* = 69, *p* < 0.0001).

### Transport of Oligomeric α-Synuclein Into Red Blood Cells *in vivo*

**FIGURE 2:**
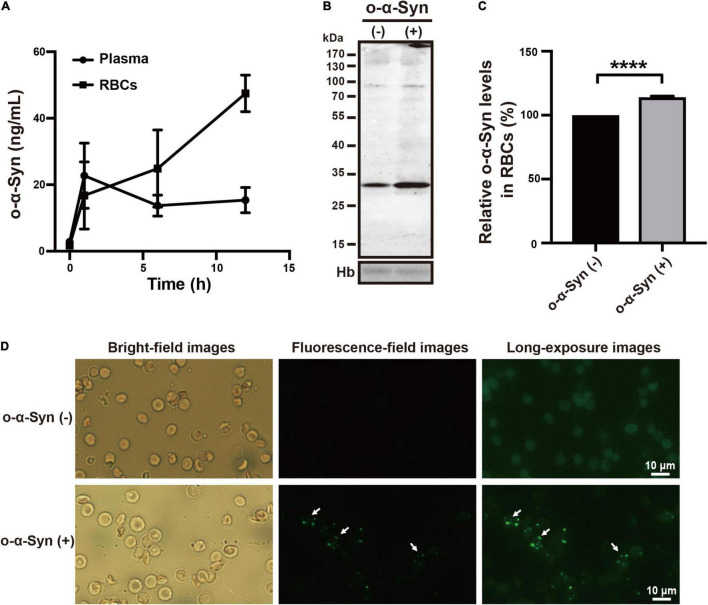
Plasma o-α-Syn transport into RBCs in mice. **(A)** RBC o-α-Syn levels in o-α-Syn-injected mice increased significantly in a time-dependent manner, contrasting with the slight change in plasma o-α-Syn levels. **(B,C)** RBCs of o-α-Syn-injected mice had more o-α-Syn than those of control mice did. **(D)** RBCs of o-α-Syn-treated mice had granular α-Syn-immunoreactive fluorescent particles (scale bar = 10 μm). *n* = 3; ****p < 0.0001. Hb, hemoglobin.

To investigate whether o-α-Syn can be actively transported from plasma into RBCs *in vivo*, we injected purified o-α-Syn (100 μL, 20 μg/mL) or the same volume of saline into the mouse tail veins. At 0, 1, 6, and 12 h after injection, venous blood was collected, plasma and RBCs were isolated, and o-α-Syn concentrations were measured. ELISA results showed that the initial concentration values of o-α-Syn in plasma and RBCs were extremely low because the endogenous mouse o-α-Syn could not be recognized efficiently by the 3D5 antibody (Li et al., 2021). Plasma o-α-Syn rapidly increased to 22.7 ng/mL during the first hour, and then gradually decreased to a relatively stable level (15.4 ng/mL). In contrast, the o-α-Syn concentration in RBCs increased to 16.8 ng/mL during the first hour, and to 47.5 ng/mL at the twelfth hour (Figure 2A). This concentration was much higher than that in plasma at the same time. Western blot analysis showed that RBCs isolated from the o-α-Syn-injected mice had increased o-α-Syn levels compared with those of control mice (Figures 2B,C). In addition, granular α-Syn-immunoreactive fluorescent particles were observed in o-α-Syn-treated RBCs but not in control RBCs (Figure 2D), further confirming the ability of RBCs to take up plasma o-α-Syn.

### Transport of Oligomeric α-Synuclein Into Isolated Red Blood Cells

**FIGURE 3:**
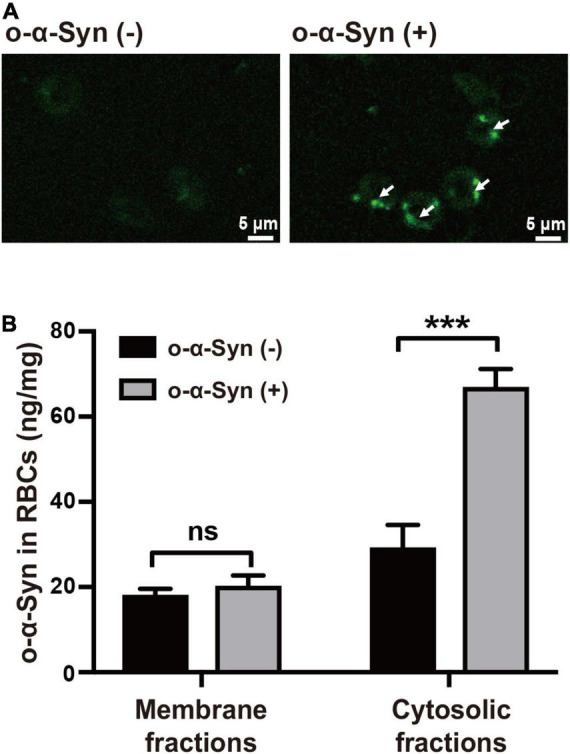
o-α-Syn transport into isolated RBCs. **(A)** Confocal microscopic analysis showed that o-α-Syn-treated RBCs had α-Syn-immunoreactive fluorescent particles in the outer region of RBCs (scale bar = 5 μm). **(B)** Significant increase in o-α-Syn levels was only detected in the cytosolic fraction and not the membrane fraction. *n* = 3; ***p < 0.001.

**FIGURE 4:**
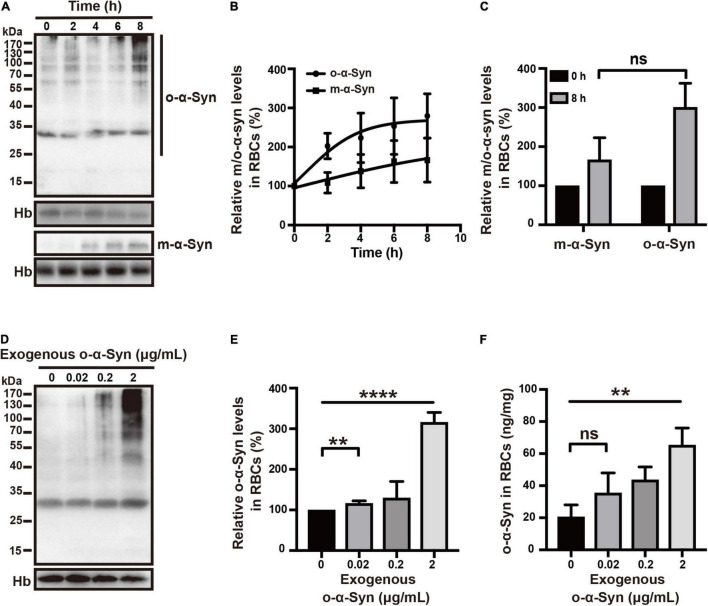
Time- and concentration-dependent changes in RBC o-α-Syn levels. **(A)** 2 μg/mL of m-α-Syn or o-α-Syn was added to RBCs incubation buffer and intracellular m-α-Syn and o-α-Syn levels were measured at different time points using Western blot. **(B)** Intracellular m-α-Syn (*r* = 0.9737, *p* < 0.01) and o-α-Syn (*r* = 0.9398, *p* < 0.05) levels increased in an incubation time-dependent manner. **(C)** Increase in o-α-Syn levels was more significant than that in m-α-Syn levels without significant difference. **(D)** Intracellular o-α-Syn levels increased with the increase in extracellularly added o-α-Syn. **(E,F)** Concentration-dependent increase in intracellular o-α-Syn. *n* = 3; **p < 0.01, ****p < 0.0001; ns, not significant. Hb, hemoglobin.

Freshly isolated RBCs were incubated at 37°C in KH buffer and 2 μg/mL of o-α-Syn was added to the buffer and incubated for different time. Confocal microscopy was used to illustrate the distribution of o-α-Syn after 8 h incubation. The α-Syn-immunoreactive fluorescent particles indicating α-Syn aggregates were seen in the outer region of RBCs but not on the membrane, suggesting the internalization of o-α-Syn (Figure 3A). Detection of o-α-Syn by ELISA also showed that significant increase in o-α-Syn levels was observed only in the cytosolic fraction (*p* < 0.001) but not the membrane fraction, further confirming that the o-α-Syn was internalized into RBCs (Figure 3B). To investigate the time-dependent intracellular accumulation of α-Syn, intracellular concentrations of m-α-Syn and o-α-Syn were measured at different time points. Increases in m-α-Syn and o-α-Syn levels were visible after 2 h (Figure 4A). Prolonging the incubation time resulted in further increases in m-α-Syn and o-α-Syn concentrations; intracellular m-α-Syn and o-α-Syn accumulation was time-dependent (*r* = 0.9737, *p* < 0.01 for m-α-Syn; *r* = 0.9398, *p* < 0.05 for o-α-Syn; Figure 4B). Moreover, translocation appeared to be more efficient for o-α-Syn than for m-α-Syn though there was no significant difference (Figure 4C).

We further examined the intracellular translocation of different concentrations of o-α-Syn (0.02, 0.2, and 2 μg/mL). Increased intracellular o-α-Syn levels were detected in RBCs treated with o-α-Syn concentrations as low as 0.02 μg/mL (Figures 4D–F). The concentrations of o-α-Syn added to KH buffer were 200 times lower than those in RBCs; the concentration increase in RBCs indicated that a certain active transport mechanism might mediate the translocation of o-α-Syn from the extracellular to intracellular space.

### Effect of Temperature on Oligomeric α-Synuclein Transport

**FIGURE 5:**
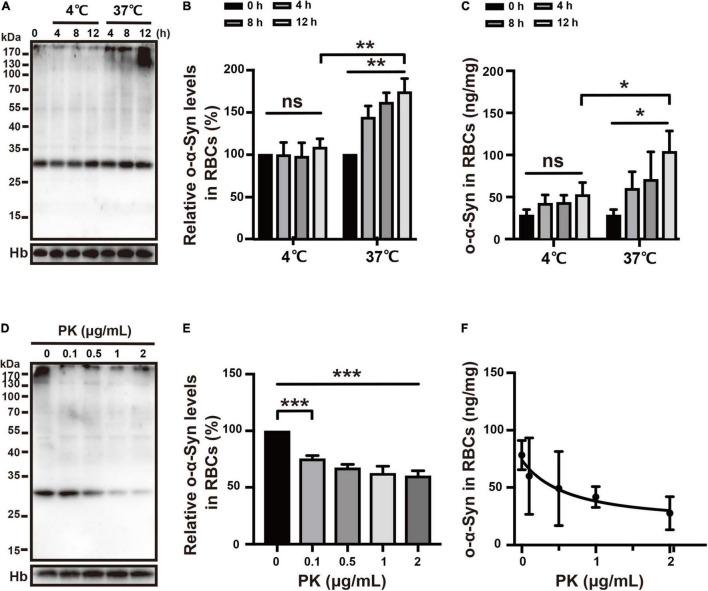
Effects of temperature and PK on o-α-Syn transport into RBCs. **(A–C)** Lowering the temperature impaired intracellular o-α-Syn accumulation after extracellular addition of o-α-Syn. **(D–F)** Pretreating RBCs with PK decreased intracellular o-α-Syn accumulation; PK concentration and intracellular o-α-Syn accumulation negatively correlated (*r* = –0.9184, *p* < 0.05). *n* = 3; *p < 0.05, **p < 0.01, ***p < 0.001; ns, not significant. Hb, hemoglobin.

RBCs use anaerobic glycolysis as an energy source. Therefore, lowering the temperature reduces glycolytic activity in RBCs and impairs the active uptake of o-α-Syn by RBCs (Guppy et al., 1992). To test this, RBCs were incubated at either 37 or 4°C and 2 μg/mL of o-α-Syn was added to KH buffer followed by incubation for different time periods. Intracellular o-α-Syn concentrations were measured using Western blot and ELISA. At 37 and 4°C, o-α-Syn was transported into RBCs, as indicated by increased o-α-Syn accumulation in o-α-Syn-treated cells. However, lowering the temperature decreased o-α-Syn accumulation in RBCs compared with that in RBCs incubated at 37°C; o-α-Syn transport was suppressed by lowering the temperature (Figures 5A–C).

### Effect of PK on Oligomeric α-Synuclein Transport

PK is often used to block membrane protein-mediated transport. To demonstrate that o-α-Syn transport was mediated by one type membrane surface protein, RBCs were pretreated with PK (0–2 μg/mL) for 10 min before 2 μg/mL of o-α-Syn was added to KH buffer. O-α-Syn accumulation was inhibited by pretreatment with PK (Figures 5D,E) and negatively correlated with PK concentration (*r* = –0.9184, *p* < 0.05; Figure 5F).

### Effects of Specific Endocytosis Inhibitors on Oligomeric α-Synuclein Transport

**FIGURE 6:**
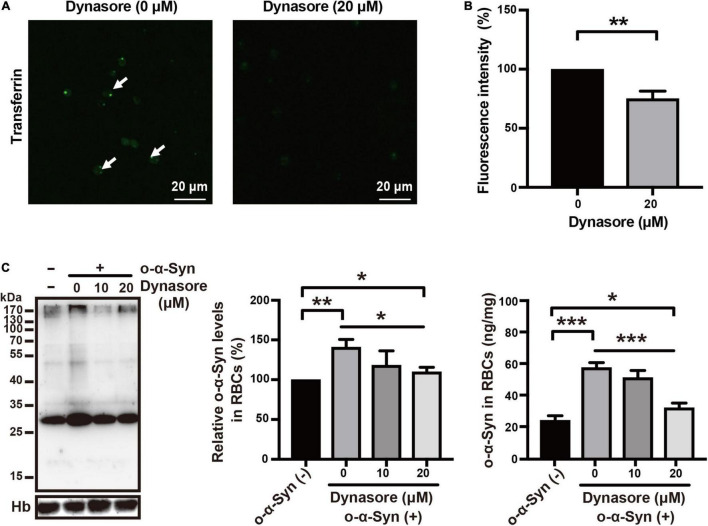
Effect of dynasore on o-α-Syn transport into RBCs. **(A,B)** RBCs were untreated or pretreated with 20 μM of dynasore, and then incubated with 10 μg/mL fluorescein-labeled transferrin for 2 h. In dynasore-treated cells, the endocytosis of fluorescein-labeled transferrin was inhibited, which was in contrast to the untreated cells. **(C)** Western blot and ELISA showing the changes of o-α-Syn levels in RBCs after pretreatment with dynasore and incubation with o-α-Syn. Intracellular o-α-Syn levels were significantly decreased in cells pretreated with dynasore. Values in non-o-α-Syn-treated cells were considered 100%. *n* = 3; *p < 0.05, **p < 0.01, ***p < 0.001, ****p < 0.0001; Hb, hemoglobin.

**FIGURE 7:**
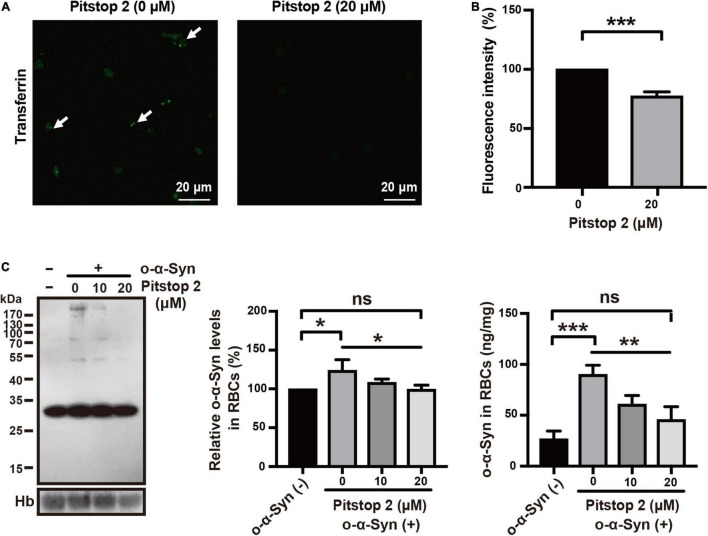
Effect of pitstop 2 on o-α-Syn transport into RBCs. **(A,B)** RBCs were untreated or pretreated with 20 μM of pitstop 2, and then incubated with 10 μg/mL fluorescein-labeled transferrin for 2 h. In pitstop 2-treated cells, the endocytosis of fluorescein-labeled transferrin was inhibited, which in contrast to the untreated cells. **(C)** O-α-Syn levels in RBCs was measured after pretreatment with pitstop 2 and incubation with o-α-Syn. Pitstop 2 decreased intracellular o-α-Syn accumulation. Intracellular o-α-Syn levels in non-o-α-Syn-treated cells were considered 100%. *n* = 3; *p < 0.05, **p < 0.01, ***p < 0.001; ns, not significant. Hb, hemoglobin.

Receptor-dependent endocytosis participates in the internalization of misfolded α-Syn aggregates; clathrin-dependent endocytosis is one of the major pathways involved in this internalization (Choi et al., 2021). We first tested the effect of dynasore, a specific dynamin inhibitor, on o-α-Syn transport into RBCs, because dynamin functions upstream of the clathrin-dependent endocytoses. The effect of the endocytosis inhibitor was confirmed by the fluorescence microscopy that showed the intensity of internalized fluorescence-labeled transferrin decreased significantly after the RBCs were pretreated with dynasore (Figures 6A,B). Western blot and ELISA results also showed the inhibitory effect of dynasore on o-α-Syn accumulation in RBCs (Figure 6C), indicating that o-α-Syn transport into RBCs was dynamin-dependent. We further examined the effects of pitstop 2, the specific inhibitor of clathrin-dependent endocytosis, on o-α-Syn transport into RBCs. Inhibitory effect was similarly confirmed in RBCs with the pretreatment of pitstop 2 using the fluorescence-labeled transferrin (Figures 7A,B). Then, significant reduction in o-α-Syn internalization were observed in pitstop 2-treated RBCs by Western blot analysis and ELISA (Figure 7C). Taken together, these results suggest that the mechanism of o-α-Syn transport into RBCs involved receptor-dependent endocytosis; in particular, clathrin-dependent pathways.

## Discussion

T-α-Syn concentration in RBCs is 1000 times higher than that in plasma (Barbour et al., 2008); however, the difference between o-α-Syn concentrations in RBCs and plasma remains unclear. Our ELISA results showed that o-α-Syn concentration in human RBCs was much higher than that in plasma. This raises the question of why RBCs accumulate much more o-α-Syn than plasma, considering that they have no nuclei and cannot synthesize α-Syn. One of the possibilities might be the translocation of o-α-Syn from plasma into RBCs.

To investigate the possibility and potential mechanism of o-α-Syn translocation from plasma into RBCs, we prepared o-α-Syn with molecular sizes ranging from 34 to 170 kDa, corresponding to dimer to decamer. We intravenously injected o-α-Syn into mice and observed the changes in o-α-Syn levels in plasma and RBCs. Mice injected with o-α-Syn had dramatically increased o-α-Syn levels in the plasma and RBCs compared with those in mice injected with saline alone. Although at the first hour, the level of o-α-Syn was higher in plasma than in RBCs, in the following time, the level of o-α-Syn in RBCs increased continuously and was much higher than that in plasma, indicating that the RBCs may enrich o-α-Syn in some active way. In addition, RBCs isolated from o-α-Syn-injected mice exhibited α-Syn-positive immunofluorescent particles similar to α-Syn aggregates, suggesting that the o-α-Syn was taken up by RBCs. To further demonstrate this transport, the extracellular o-α-Syn at concentration much lower than that in RBCs was incubated with isolated human RBCs, and its translocation from extracellular space to intracellular space was investigated. Confocal microscopic analysis revealed that o-α-Syn-immunoreactive signals were distributed on the outer region of the RBCs and not on the membrane. Considering that RBCs are double concave disc shape and there is less cytoplasm in the center of the cells, the above distribution suggests that o-α-Syn is localized in the inside of the cells. This localization was confirmed by ELISA analysis of o-α-Syn in the cytosolic and membrane fractions, which showed that o-α-Syn was only detected in the cytosolic and not membrane fractions. The above results indicate that the extracellularly added o-α-Syn was transported into the inside of RBCs. In addition, this o-α-Syn transport was time- and concentration-dependent. The above results suggest that a certain active transport mechanism might participate in o-α-Syn uptake by RBCs.

Several pathways have been reported regarding the internalization of extracellular α-Syn. Extracellular m-α-Syn penetrates into live cells via passive diffusion and in a temperature-insensitive manner (Ahn et al., 2006). Transmembrane transport of m-α-Syn is aided by its N-terminal KTVEGV repeats and is not affected by endocytosis inhibitors (Ahn et al., 2006; Park et al., 2009). However, passive diffusion is not effective for the intracellular translocation of aggregated α-Syn. It has been reported that the internalization of α-Syn fibrils can be mediated by macropinocytosis and receptor-dependent endocytosis (Delenclos et al., 2017; Bieri et al., 2018). The internalization of α-Syn oligomers is usually mediated by receptor-dependent endocytosis; in particular, clathrin-dependent endocytosis (Valdinocci et al., 2017). Although endocytosis has rarely been studied in RBCs, there are a few reports showing the presence of receptor-dependent endocytosis in RBCs (Schrier et al., 1979; Colin and Schrier, 1991). We therefore speculated that α-Syn oligomers might be internalized into RBCs by receptor-dependent endocytosis, in particular the clathrin-dependent endocytosis as demonstrated in other cells. Since receptor-dependent endocytosis are sensitive to temperature and proteolytic enzymes (Harding et al., 1983; Larrick et al., 1985), we therefore observed the effects of lowering the temperature and PK treatment on o-α-Syn internalization. Under both conditions, o-α-Syn accumulation in RBCs drastically decreased after the extracellular addition of o-α-Syn. Moreover, intracellular o-α-Syn accumulation was inhibited by pretreating RBCs with specific inhibitors of receptor-dependent endocytosis, including dynasore and pistop 2. Dynasore is a specific inhibitor of dynamin which functions upstream of multiple receptor-dependent endocytic pathways, including clathrin-dependent pathways (Macia et al., 2006; Doherty and McMahon, 2009). Pistop 2 is specific inhibitors of clathrin-dependent endocytosis (Robertson et al., 2014; Horibe et al., 2018). Based on the above results, we speculate that one of mechanisms that mediates the transport of o-α-Syn from plasma into RBCs is clathrin-dependent endocytosis, although other potential mechanisms such as caveolae-dependent endocytosis could not be excluded.

Increased o-α-Syn levels in RBCs have been reported in patients with PD and MSA (Wang et al., 2015; Papagiannakis et al., 2018; Tian et al., 2019); detection of RBC o-α-Syn is a promising diagnostic biomarker for PD and MSA. Because brain α-Syn can cross the blood-brain barrier and be transported into the blood circulation, o-α-Syn entering blood plasma can be further transported into RBCs, leading to o-α-Syn accumulation in RBCs. Therefore, the detection of RBC o-α-Syn as a biomarker could be an ideal alternative strategy to predict o-α-Syn changes in the brain without hemolysis interference. Another advantage of detecting RBC o-α-Syn is that the signal could be amplified due to the ability of RBCs to accumulate o-α-Syn, as demonstrated by our *in vivo* experiments.

The present study has some limitations. First, we used non-biotinylated and biotinylated 3D5 monoclonal antibodies for ELISA detection. Because the antibody mainly recognizes human α-Syn and only weakly reacts with mouse α-Syn, the endogenous o-α-Syn in mouse RBCs was less detected with this antibody, making the basal value of o-α-Syn measured in the mouse RBCs much lower than that in human RBCs. In addition, although the injection of exogenous o-α-Syn led to continuous increase in o-α-Syn levels in mouse RBCs during the observed 12 h, it had not reached the peak level. This may be another reason that the measured internalized o-α-Syn level in mouse RBCs was lower than that in human RBCs. Second, the present study only studied the role of clathrin-dependent endocytosis in the o-α-Syn transport from plasma to RBCs, there are other potential pathways remain to be investigated. Third, specific membrane receptors that mediate the translocation have not been identified.

In summary, the present study provides evidence that o-α-Syn could be actively transported from plasma into RBCs through receptor-dependent endocytic pathways. Clathrin-dependent endocytosis might be one of the major pathways that mediates o-α-Syn translocation. Further studies are needed to provide direct evidence for the link between changes in o-α-Syn levels in the brain and RBCs.

## Data Availability Statement

The original contributions presented in the study are included in the article/supplementary material, further inquiries can be directed to the corresponding authors.

## Ethics Statement

The studies involving human participants were reviewed and approved by Institutional Review Board and Ethics Committee of Xuanwu Hospital, Capital Medical University. The patients/participants provided their written informed consent to participate in this study. The animal study was reviewed and approved by Institutional Animal Care and Use Committee of Xuanwu Hospital.

## Author Contributions

SY and CW took responsibility for the study design and the edit of final version of the manuscript. WL was responsible for designing experiments, performing, and drafting the manuscript. JH conducted the experiments and analyzed data. XiL, XuL, and ZL were in charge of technical and material support. All authors read and approved the final manuscript.

## Conflict of Interest

The authors declare that the research was conducted in the absence of any commercial or financial relationships that could be construed as a potential conflict of interest. The reviewer JL declared a shared parent affiliation with the authors to the handling editor at the time of review.

## Publisher’s Note

All claims expressed in this article are solely those of the authors and do not necessarily represent those of their affiliated organizations, or those of the publisher, the editors and the reviewers. Any product that may be evaluated in this article, or claim that may be made by its manufacturer, is not guaranteed or endorsed by the publisher.
